# Unlocking Bio-Instructive Polymers: A Novel Multi-Well Screening Platform Based on Secretome Sampling

**DOI:** 10.21769/BioProtoc.4939

**Published:** 2024-02-20

**Authors:** Shirin Fateh, Reem A. Alromaihi, Amir M. Ghaemmaghami, Morgan R. Alexander

**Affiliations:** 1 School of Pharmacy, University of Nottingham, Nottingham, UK; 2 School of Life Sciences, University of Nottingham, Nottingham, UK

**Keywords:** Biomaterials, High-throughput screening, HTS, Secretome profiling, Immune-instructive, Bio-instructive, 2D printing, Polymer microarray

## Abstract

Biomaterials are designed to interact with biological systems to replace, support, enhance, or monitor their function. However, there are challenges associated with traditional biomaterials’ development due to the lack of underlying theory governing cell response to materials’ chemistry. This leads to the time-consuming process of testing different materials plus the adverse reactions in the body such as cytotoxicity and foreign body response. High-throughput screening (HTS) offers a solution to these challenges by enabling rapid and simultaneous testing of a large number of materials to determine their bio-interactions and biocompatibility. Secreted proteins regulate many physiological functions and determine the success of implanted biomaterials through directing cell behaviour. However, the majority of biomaterials’ HTS platforms are suitable for microscopic analyses of cell behaviour and not for investigating non-adherent cells or measuring cell secretions. Here, we describe a multi-well platform adaptable to robotic printing of polymers and suitable for secretome profiling of both adherent and non-adherent cells. We detail the platform's development steps, encompassing the preparation of individual cell culture chambers, polymer printing, and the culture environment, as well as examples to demonstrate surface chemical characterisation and biological assessments of secreted mediators. Such platforms will no doubt facilitate the discovery of novel biomaterials and broaden their scope by adapting wider arrays of cell types and incorporating assessments of both secretome and cell-bound interactions.

Key features

• Detailed protocols for preparation of substrate for contact printing of acrylate-based polymers including O_2_ plasma etching, functionalisation process, and Poly(2-hydroxyethyl methacrylate) (pHEMA) dip coating.

• Preparations of 7 mm × 7 mm polymers employing pin printing system.

• Provision of confined area for each polymer using ProPlate^®^ multi-well chambers.

• Compatibility of this platform was validated using adherent cells [primary human monocyte–derived macrophages (MDMs)) and non-adherent cells (primary human monocyte–derived dendritic cells (moDCs)].

• Examples of the adaptability of the platform for secretome analysis including five different cytokines using enzyme-linked immunosorbent assay (ELISA, DuoSet^®^).

Graphical overview

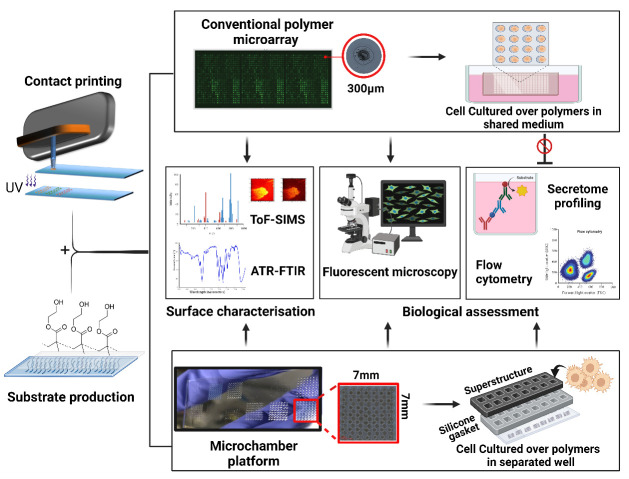

## Background

Biomaterials play a pivotal role in the development of advanced medical devices, drug delivery systems, and regenerative therapies to enhance patient outcomes and quality of life [1–4]. However, current drawbacks include device-associated infection, adverse immune responses, and in-service degradation that can collectively reduce implant performance [5–7]. Rational design of bio-instructive materials remains unattainable due to our limited understanding of material–biological interactions [1]. As a result, screening is a commonly employed approach for discovering and optimising novel biomaterials with a desired biological function, such as pro- or anti-inflammatory properties. High-throughput screening (HTS) strategies have accelerated the development of biomaterials by enabling researchers to rapidly analyse a large number of samples or conditions in a systematic manner [8]. A commonly used HTS approach for biomaterials is the use of printed polymer microarrays, which have been employed to screen cell responses to materials, allowing reproducible control over cellular behaviour [9]. Key examples are screening for scalable synthetic cultureware for human pluripotent stem cells [10], polymers to modulate the foreign body response and wound healing [11,12], and a new class of bacteria-attachment-resistant materials [13]. In these studies, thousands of materials are robotically printed and in situ ultraviolet (UV)-polymerised on a single slide. This approach is used to discern specific biological interactions by culturing cells directly on the polymer array surfaces within a shared culture medium [14,15]. Despite the success of such polymer microarray platforms in biomaterials’ discovery, the secreted biochemical signals are an untapped resource in understanding cellular phenotypes. Paracrine signalling is also a worry requiring follow-up studies on individually scaled up polymers before potential hits can be verified. Understanding cell-surface interactions requires probing biochemical cues, in addition to biomechanical, topographical, and material chemistry/bio-interfacial cues. Hence, without knowledge of the secretome profile of the cells following their interaction with materials, the understanding of the functionality of the polymer is incomplete. Furthermore, polymer microarrays are limited to the assessment of cells with strong adhesion abilities. Notably, for cells such as dendritic and T cells, which possess weaker substrate adhesion tendencies, attributing their phenotypical changes to a particular polymer spot would be incredibly challenging [16–18].

In response to these limitations of the polymer microarray, we developed a novel platform that combines existing arraying technology such as contact and inkjet printing, multi-well microchambers, and high-throughput secretome profiling. The platform benefits from reusable superstructures (ProPlate^®^) that are mountable on the printed glass substrates. This offers the provision of confined culture volumes, each designated for a distinct polymer condition with a surface area and volume compatible with cell culture for a few days. This separation allows us to closely study the mediators released by cells, helping us understand how these materials interact with cells and the mechanisms of the biomolecules involved. In addition, this system allows for high-throughput surface characterisation such as time-of-flight secondary ion mass spectrometry, x-ray photoelectron spectroscopy, and attenuated total reflectance–Fourier transform infrared spectroscopy (ATR-FTIR), ensuring the accurate identification and validation of controlling moieties [19]. Together, these analytical approaches have the potential to enhance the discovery of new biomaterials and improve our understanding of biomaterials–cells interface. Yet, there are constraints to the throughput and time-related aspects of this platform. In a single operational cycle, the platform demonstrates the capacity to fabricate ten slides, each accommodating 16 unique chemical compositions, resulting in the synthesis of 160 distinct polymer entities, thereby yielding 8,960 discrete polymer spots. By way of comparison, a polymer microarray system can generate 17,280 polymer deposition sites from 576 chemistries in triplicates across ten slides [20]. Nevertheless, this limited output can be ameliorated through the implementation of 64-well superstructures from GraceBio-Labs and the application of multiplexing methodologies as alternatives to conventional techniques such as enzyme-linked immunosorbent assay (ELISA). Here, we provide a step-by-step description of methodologies and troubleshooting aspects involved in developing this platform, including preparation of substrate, fabrication of multi-well chambers, and provision of confined area for each polymer condition, followed by relevant examples of the anticipated outcomes such as chemical characterisation and biological assessments of the secreted soluble mediators.

## Materials and reagents

**Reagents**

1. Oxygen (O_2_) gas (any vendor)
2. Molecular sieves (4 Å) (VWR International, catalog number: 215-283-8)
3. Toluene (Fisher Scientific, catalog number: T290-4)
4. 3-glycidoxypropyltrimethoxysilane (GPTMS) (Sigma-Aldrich, catalog number: 440167-500mL)
5. Argon gas (any vendor)
6. Acetone (Fisher Scientific, catalog number: A949SK-4)
7. Poly(2-hydroxyethyl methacrylate) (pHEMA) (Sigma-Aldrich, catalog number: P3932-25g)
8. Deionised distilled water (Milli-Q^®^) (Millipore, Sigma-Aldrich, USA)
9. Ethanol (Sigma-Aldrich, catalog number: 1009862500)
10. Ethoxyethyl acrylate (EOEA) (Sigma-Aldrich, USA, catalog number: 106-74-1)
11. Dimethylformamide (DMF) (Fisher Scientific, catalog number: AA22915K7)
12. Photoinitiator (2,2-dimethoxy-2-phenylacetophenone) (DMPA) (Sigma-Aldrich, catalog number: 19611-8)
13. Isopropanol (Sigma-Aldrich, catalog number: 563935)
14. Tween^®^ 20 (Sigma-Aldrich, catalog number: 9005-64-5)
15. Phosphate buffer solution (PBS) (Sigma-Aldrich, catalog number: D8537)
16. Trypan blue dye (any vendor)
17. ToxiLight^TM^ assay (Lonza, USA, catalog number: LT17-217)
18. ELISA DuoSet^®^ (R&D Systems, USA) (TNF-α, catalog number: DY210; IL-10, catalog number: DY217; TGF-β1, catalog number: DY240; CCL-18, catalog number: DY394; IL-6, catalog number: DY206; IL-12, catalog number: DY1270)

**Laboratory supplies**

1. Glass slides (25 mm × 75 mm) (VWR, catalog number: 631-1553)
2. Glass beaker (any vendor)
3. Needles (21 G, 120 mm) (Fisher Scientific, catalog number: 10438881)
4. Needles (21 G, 40 mm) (Sterican^®^ Safety Needle, catalog number: Z118044)
5. Plastic syringe 50 mL (any vendor)
6. Crystallizing dish (Pyrex, capacity 1,200 mL)
7. Parafilm^®^ (Sigma-Aldrich, catalog number: P7543)
8. Falcon tube 50 mL (Sigma-Aldrich, catalog number: T2318)
9. Polypropylene 384-well plate (Corning, product number: 3656)
10. Glass Pasteur pipette (VWR, catalog number: 612-1702p)
11. Weighing boats (any vendor)
12. Glove box (MBRAUN, Germany)
13. Microarray print head 16 pins (BioDot, USA)
14. Plastic snap clips (GraceBio-Labs, catalog number: 204830)
15. 4-well rectangular plate for slides (Thermo Fisher, Nunc^TM^, catalog number: 267060)

## Equipment

1. Glass funnel (any vendor)
2. Support stand (A-frame) (any vendor)
3. Laboratory clamp (any vendor)
4. Hotplate (any vendor)
5. Stainless steel rack for glass slides (Sigma-Aldrich, catalog number: Z710989)
6. Fume hood (any vendor)
7. Plasma etcher (Diener, model: Nano LFG40)
8. Vacuum oven (Thermo Scientific, model: Vacutherm)
9. Sonicator (any vendor)
10. Dip-coater (Holmarc, model: HO-TH-01 dip-coater)
11. Water contact angle measurement apparatus (KSV Instruments, model: CAM 100)
12. Polypropylene pipette tips (any vendor)
13. Pipette (any vendor)
14. Electric pipettor controller (any vendor)
15. Weighing scale (0.01 g and 0.0001 g sensitivity) (any vendor)
16. Spatula (any vendor)
17. Pin printing workstation (BioDot, model: XYZ3200)
18. Microarray ceramic pin 500 µm (LabNEXT Inc, model: Xtend^TM^)
19. UV lamp (365 nm, any vendor)
20. O_2_ sensor (Cambridge Sensotec, model: rapidox 1100)
21. Optical profiler (KLA, model: Zeta^TM^-300)
22. Multi-well chambers (16-well ProPlate^®^) (GraceBio-Labs, catalog number: 244864)

## Software and datasets

1. BioDot AxSys^TM^ (BioDot, USA)
2. MicroLab expert (Agilent, USA)
3. Spectrus Processor (ACD lab, USA)
4. GraphPad Prism software (Version 10.0.2, USA)

## Procedure

This method consists of three key steps: O_2_ plasma etching, optimisation of functionalisation process, and pHEMA dip coating. Dip coating of glass substrate with pHEMA was performed as previously described [20] with some modifications as detailed here.

**Part I. Substrate preparation**

- **O_2_ plasma etching**
  1. Put conventional 25 mm × 75 mm glass sides in plasma etcher chamber and subject them to O_2_ plasma (P = 300 mbar, 100 W) for 10 min.
  2. To confirm that the slides are activated by excited ions, measure water contact angle (WCA) before and after plasma treatment [21]. An example is shown in Figure 1; the presence of organic contamination increases the water contact angle; therefore, its removal results in a reduction in the observed contact angle [22,23].
- **Functionalisation process**
  1. Add molecular sieves (4 Å) to toluene at 20% w/v for 24 h prior to the silanisation process to have anhydrous toluene. See Troubleshooting.
  2. After the etching step, submerge glass slides immediately into 500 mL of anhydrous toluene in a crystallisation dish.
  3. Place the reaction vessel on a hot plate set to 50 °C (see Troubleshooting).
  4. Put the reaction under an argon atmosphere, place the funnel over the reaction vessel, and connect the argon to the neck of the funnel.
  5. Add 10 mL of GPTMS into the anhydrous toluene solution.
  6. Allow this reaction to proceed for 24 h to achieve completion. You can use a frame stand and clamp to secure the reaction and argon.
  7. Cool the slides to room temperature and wash them three times in fresh acetone to remove any residue of unbound silane.
  8. Dry the slides under vacuum (< 50 mTorr) for 24 h.
  9. Use WCA measurement after the functionalisation step to ensure that silanisation has taken place completely. The functionalised glass slide becomes hydrophobic with a higher contact angle of 54° ± 1.11 (Figure 1). Measuring the WCA on a substrate is a commonly used method to quantify the substrate wettability and its cleanliness.
  **Figure 1.**
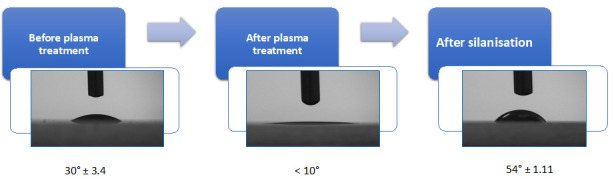
Quality control for the effectivity of plasma etching and silanisation process. Water contact angle (WCA) measurement of the glass substrate to validate the functionalisation. The O_2_ plasma–activated glass slide shows increased hydrophilicity with very low contact angle of < 10°, while after silanisation the substrate becomes hydrophobic with a higher contact angle of 54° ± 1.11.
- **pHEMA dip coating**
  1. Prepare 4% (w/v) pHEMA solution in ethanol (95% v/v in deionised distilled water). See Troubleshooting.
  2. Set up the settings of the dip-coater to 9 mm/s speed and a dip duration of 2 s with a retention speed of 1 s.
  3. Clip the glass slides to the holder and adjust the holder position to a proper height vertically.
  4. Pour pHEMA solution in a 100 mL beaker and immerse epoxy silanised glass slides in the 4% pHEMA solution.
  5. Repeat coating four times with enough time for drying in between the dips (see Troubleshooting). While waiting for the slides to dry, cover the beaker containing pHEMA to prevent evaporation.
  6. Leave the pHEMA-coated slides at atmospheric conditions for three days prior to its use for arraying.
  7. Use optical profilometry to evaluate the evenness of pHEMA coating of the glass substrates. Figure 2 illustrates the uniformity of step height results from pHEMA coating.
  **Figure 2.**
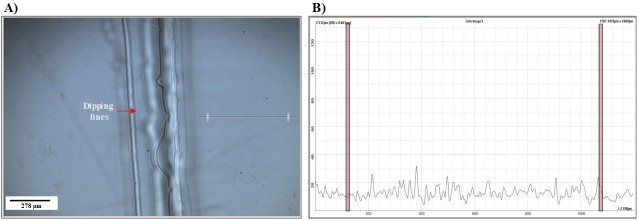
Evaluation of evenness of pHEMA coat. Optical profilometer images and height line scans (5×) of a pHEMA-coated glass slide. (A) 2D image of the pHEMA-coated glass slide. The arrow shows the measured distance; the dipping lines are apparent on the side of the glass substrate, as the glass is coated four times. (B) Graph representing the space highlighted on the 2D image (A) showing the evenness of the pHEMA coat, as the step height variation is minimal.

**Troubleshooting**

1. Plasma treatment has been proven to be a simple technique for modifying surface properties, ensuring removal of impurities and organic contaminants from the surface, which are fixed by weak electrostatic/van der Waal’s forces [24]. Bombardment of plasma excited ions to the surface of the glass slide promotes hydroxylation (OH groups) and generates the Si–OH groups.
2. Excess amount of water is detrimental to the extent of silanisation; therefore, use of molecular sieves in proper proportions to the water content of the solvent is critical [22].
3. Maintaining the reaction at 50 °C is crucial to lower the number of weakly bonded silane molecules by disrupting the hydrogen bonds in silane layer [25].
4. The silane provides a bind between the glass slide and the pHEMA coat, as the methoxy groups of GPTMS bind the -OH on the glass slide and the reactive epoxy group binds -CH in pHEMA [22,26].
5. To speed up the dissolvability of pHEMA in the intended solvent, it is useful to use smaller amounts in larger containers and sonication. For example, add 2 g of pHEMA in 50 mL of 95% ethanol/deionised distilled water, close the cap, and leave the tube in the sonicator for 24 h. Ultrasonication is often used to promote an effective and fast dissolution of pHEMA powder in ethanol.
6. Wet pHEMA appears cloudy on the surface of the glass slide and it takes much longer to dry between coating repetitions.
7. Tight sealing containers for pHEMA solutions are very important, as evaporation causes concentration change. Therefore, it is best to prepare fresh pHEMA solution for each coating session.
8. Coating the glass substrate with pHEMA provides an anchorage for arraying a diverse acrylate and methacrylate polymer library [4,9]. As pHEMA swells in the presence of atmospheric moisture, it provides the possibility to form an interpenetrating network between the deposited polymer spots and the pHEMA. This increases their stability in the pretreatment washing steps, sterilisation process, and cell culturing conditions.

**Part II. Fabrication of polymer wells**

- **Preparation of polymerisation solution**
  1. Degas monomers under argon for 30 min by purging method.
    - First, add the required amount of monomer in a scintillation glass vial and seal the cap using rubber stoppers.
    - Add a layer of parafilm around the cap area to ensure that the vial is airtight.
    - Insert a short needle (21 G, 40 mm) through the cap to enable the degassing process.
    - Insert a longer needle (21 G, 120 mm) to bubble argon through the monomer. Make sure that the needle attached to argon source is dipped in the monomer while the degassing needle is away from the monomer.
  2. Weigh DMPA and make 2% (w/v) in DMF in a polypropylene or glass vial.
  3. Prepare the polymerisation solution by mixing 50% (v/v for liquids, w/v for solids) monomer in DMF and 1% DMPA in a glove box under argon condition with O_2_ levels < 2,000 ppm. See Troubleshooting.
  4. Transfer 40 μL of each polymerisation solution to a source plate (e.g., 384-well polypropylene plate).
  5. Position the source plate in its place on printing machine stage.
- **Polymer deposition and in situ UV curing**
  1. Set the printing conditions inside the chamber for O_2_ < 2,000 ppm using argon and 30%–40% humidity. See Troubleshooting.
  2. Wash the pin with isopropanol and insert into the microarray print head.
  3. Close the printer chamber to keep the printing environment settings.
  4. Initiate the software (BioDot AxSys^TM^) for printer stage and head. Recalibrate the X, Y, and Z positioning. Refer to Table 1 for the instrumental factors including print head travel speed and contact times.
    **Table 1.** Instrumental settings for movements of the stage and pin holder

Loading sample from source plate	
Held in monomer solution	Withdrawal speed
4 s	25 mm/s
**Monomer solution deposition on the substrate**	
Total contact time for each contact	Withdrawal speed
10 ms	175 mm/s
**Z-axis speed**	
6.531 mm/s	
**Vacuum wash interval**	
Held in vacuum	Withdrawal speed
10 s (3×)	175 mm/s
  5. Pre-spot four times on a plasma-etched glass slide prior to deposition of the polymerisation solution on pHEMA-coated substrate. The blotting pattern can be two contacts per position to remove the excess polymerisation solution from the outside of the pin.
  6. Start the printing process. For example, for an area of 7 mm × 7 mm of pHEMA-coated glass slide to be coated by acrylate polymers, print 56 spots with 850 μm distance in the y-axis, with an alternating +850 μm/-850 μm offset in the x-axis. Figure 3A illustrates the brightfield images of the single printed polymer spots.
  7. Polymerise deposited spots with 40 s of long wave UV (365 nm) intervals to liberate the radical ions in the DMPA, which creates the polymerisation of monomers. During this time, the pin is washed in DMF and dried three times before printing the next polymer.
  8. After printing all the polymers, further add 20 min of UV exposure at the completion point of each printing session.
  9. Keep the printed glass slides in < 50 mTorr vacuum for 24 h prior to the second print in between spots.
  10. To print in between polymer spots for full coverage, increase the initiation position by 800 μm. Figure 3B shows the double-printed spots coverage.
    **Figure 3.**
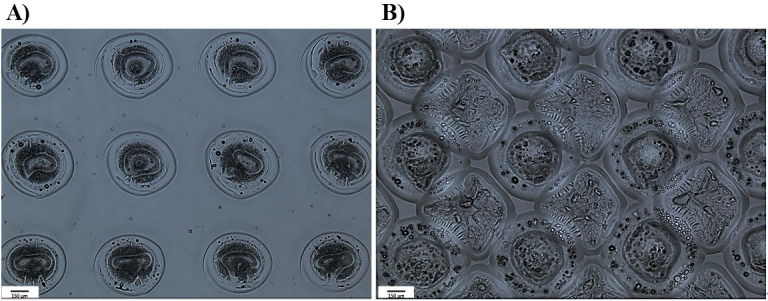
Comparison of brightfield (5×) images of printing pattern. A. Single-printed polymer spots. B. Double-printed polymer surface. The visual examination demonstrates a significantly enhanced substrate coverage after the second printing.
  11. Repeat step B6.
  12. Lastly, keep the printed slides in < 50 mTorr vacuum for at least seven days to ensure extraction of the residual solvent and unpolymerised monomers.

**Troubleshooting**
1. Be vigilant about potential issues arising from phase separation before printing, possibly linked to solvent compatibility.
2. Minimise contamination in polymerisation solutions by using solvent-compatible pipetting tools. For example, avoid using polystyrene pipetting tips while using DMF as a solvent.
3. There are many aspects to consider in contact printing to ensure consistency of the polymer areas; some of these are addressed through optimisation of the chamber environment such as humidity, temperature, and O_2_ percentage. O_2_ higher than 2,000 ppm inhibits radical polymerisation; 30%–40% humidity reduces the static effects of printing head movement and induces swelling in pHEMA to ease the interpenetration of the formed polymer spot [9,20,27].
4. Even though pHEMA provides the possibility to form an interpenetrating network between the deposited polymer spots and is known to be a low fouling material [28], it has been observed that it has effects on cell biology [29,30]. This could influence the interpretation of cells’ behaviour towards the printed polymers as it decreases the signal-to-noise ratio. This can be resolved by double-printing in between the spots.
5. For this work, inert ceramic capillary pins were used for polymer deposition using contact printing technique. Figure 4A is the schematic of the contact printer robotic head and printing steps. Ultra-smooth surface and tubular construction of these pins eliminates cross-sample contamination and improves spot uniformity and deposition repeatability while printing a large library of chemistries [31].
6. Optimise the printing pattern via trialling with spot–spot spacing (refer to Figure 4B) in order to achieve a coalescence free coverage of the surface by deposited polymer spots.
7. Consider volatility of the polymerisation solutions to adjust timing of the printing.
8. It is worth mentioning that this platform has the flexibility to utilise inkjet printing as an alternative to contact printing. Trials for proof of concept have been successfully carried out, involving spot pattern adjustments, voltage, pulse, and uniformity. This approach is particularly advantageous for printing on sensitive and/or expensive surfaces.
  **Figure 4.**
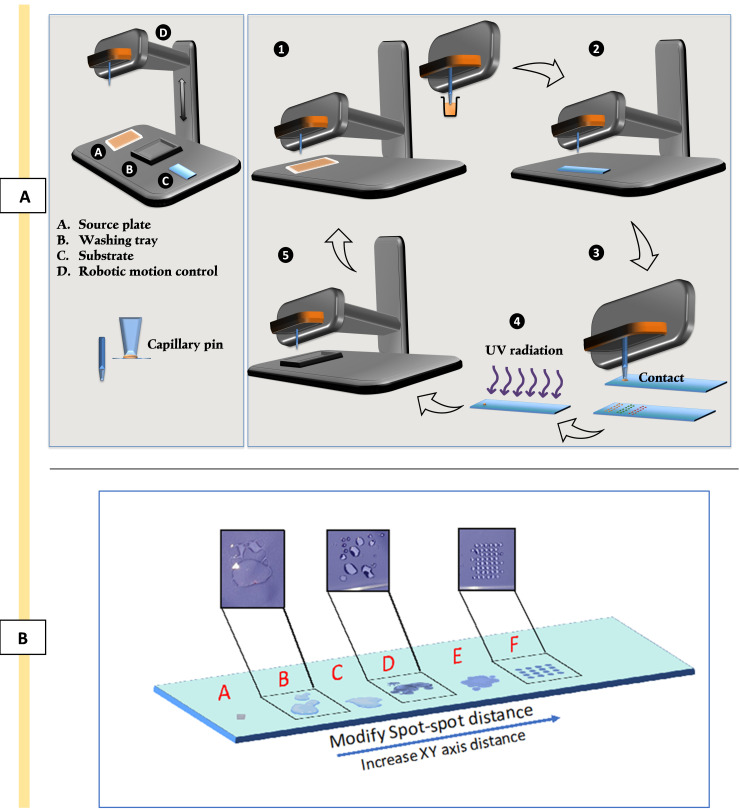
Schematic representation of contact printing technique and optimising printing patterns. A) The process of contact printing: 1) picking up the polymerisation solution from the source plate; 2) movement of the robotic head to the position coordination; 3) deposition of polymerisation solution via contacting the surface of the substrate; 4) in situ polymerisation by exposure to UV; 5) washing and vacuuming of the pin for the next material. B. Optimising printing patterns to address coalescence issues. Image shows trials B, C, D, and E and achieving uniform thin polymer layers (spot F) by minimising coalescence via xy-axes adjustments.

**Part III. Provision of confined area for each polymer**

- **Preparation of the microchambers**
  ProPlate^®^ are reusable and should be thoroughly cleaned before and after each experiment to remove any dust, particles, or contaminants, following the instructions provided by the manufacturer and summarised below:
  1. Make a washing solution by adding 0.1% (v/v) Tween^®^ 20 to PBS.
  2. Soak the chamber and clips in a clean container for 15 minutes in the washing solution.
  3. Rinse three times with deionised distilled water.
  4. Soak in isopropanol overnight.
  5. Air dry before use.
- **Leakage assessment**
  Conduct a preliminary leakage assessment to verify the integrity of the superstructure assembly, ensuring the absence of cross-contamination or leakage between the individual wells. The efficacy of the plates in retaining samples within the designated wells was qualitatively evaluated by adding trypan blue dye into the wells in an alternating manner (refer to Figure 5) and subsequently incubating them for a period of seven days or in accordance with the experimental timeline.
  **Figure 5.**
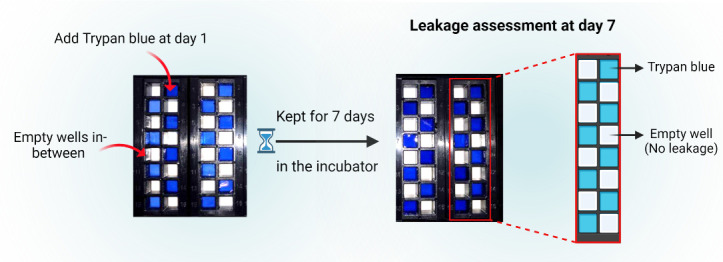
ProPlate^®^ leakage assessment. Evaluate sample retention by adding trypan blue dye to alternating wells and incubate for seven days or as per the experiment timeline to confirm the superstructure's integrity.
- **Assembly of the multi-well chambers on the printed polymer slides**
  This platform integrates contact printing microarray technology with high-throughput microtiter plate processing. Slides with polymer squares were assembled using multi-well structures to provide enclosed areas for each polymer with a total of 16 per slide. Figure 6 represents schematically the steps for mounting the multi-well chambers.
  **Figure 6.**
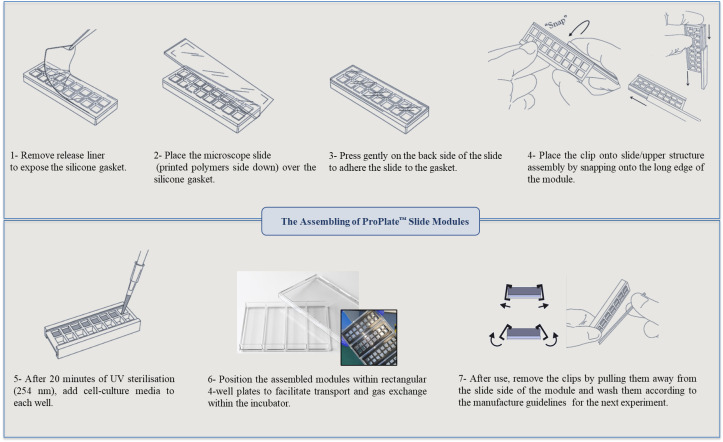
Assembly steps for the multi-well chambers on printed polymer slides. Images of the ProPlate^®^ have been adapted from GraceBio-labs website [32].

## Data analysis

ATR-FTIR spectra were acquired from MicroLab expert (Agilent, USA) and analysed by Spectrus Processor. Results for viability were measured indirectly by performing a cytotoxicity assay and presented as a percentage of the control (in this work, the control was commercial TCP). The corresponding concentrations of cytokines were measured by extrapolating the colorimetric readings (O.D.) to standard curve ELISA from three independent experiments each with two technical replicates. For both viability and secretome data, GraphPad Prism software was used to plot the graphs as mean and standard deviation (SD).

## Validation of protocol

Presence of polymers (fabricated using this platform) on the surface of the substrate was validated by ATR-FTIR. The applicability of this platform for cell culture was validated by culturing two different primary cell types and assessing cell viability and cytokine secretion:

1. **Chemical characterisation:** ATR-FTIR spectra was used to validate the presence of specific chemical components or functional groups on the substrate surface following contact printing and in situ UV polymerisation. Spectra were obtained by scanning the samples (254 scans) from 600 to 4,000 cm^-1^ with a 4 cm^-1^ resolution, with subtraction against background (air) after each scan. The analysis of the printed polymers' spectra reveals significant distinctions when compared to pHEMA. Specifically, the O–H stretching (3,550–3,200 cm^-1^) present in the pHEMA spectra is no longer evident, and distinct new peaks have emerged in the polymer spectrum in the fingerprint region (400–1,500 cm^-1^). For example, the ATR-FTIR analysis clearly confirms the conversion of ethoxyethyl acrylate (EOEA) monomer into a polymer (pEOEA). The disappearance of the C=C stretching peak at 1,640 cm^-1^, characteristic of the monomer, in the polymer spectrum, along with the presence of typical acrylate polymer peaks (e.g., C=O stretching at 1,720 cm^-1^), indicates successful polymerisation as shown in Figure 7A and B.
  **Figure 7.**
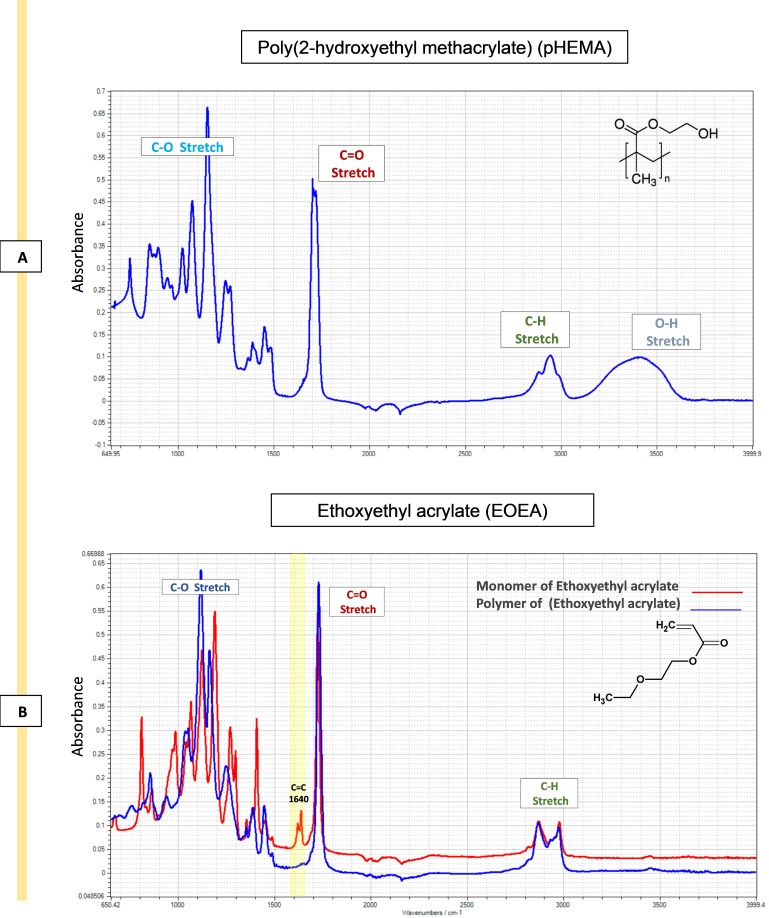
Attenuated total reflectance–Fourier transform infrared spectroscopy (ATR-FTIR) spectra of pHEMA and ethoxy ethyl acrylate (EOEA) monomer (red) and its corresponding printed polymer (blue). A. The spectra of pHEMA shows O–H stretching (3,550–3,200 cm^-1^), C–H stretching, corresponding aliphatic stretching vibration of CH_2_ (3,000–2,840 cm^-1^), and C=O stretching (1725 cm^-1^). B. The disappearance of the 1,640 cm^-1^ peak in the polymer spectrum (blue) indicates that the C=C double bond in the EOEA monomer has polymerised, leading to structural changes. Characteristic peaks at 1,720 cm^-1^ (C=O ester group) and 2,960/2,880 cm^-1^ (C-H groups) remain. Also, O–H stretching (3,550–3,200 cm^-1^) peak of pHEMA structure does not appear in pEOEA spectra, indicating the full coverage of the surface.
2. **Viability assessment:** Cell viability was assessed after 24 h of incubation of cells on the surface of the polymers using ToxiLight^TM^ assay by measuring the release of adenylate kinase from damaged cells. To showcase the applicability of the platform, viability results were measured for two distinct cell types: monocyte-derived dendritic cells (moDCs) and monocyte-derived macrophages (MDMs). The polymers fabricated through this platform support cell viability equal or higher than tissue culture plastic control (TCP), as illustrated in Figure 8.
  **Figure 8.**
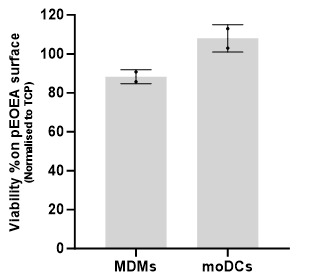
Viability assessment. Two distinct primary cell types were examined following a 24 h incubation with pEOEA using the microchamber platform. The graphs depict the mean ± SD results from two independent experiments, demonstrating that the polymer supports cell viability. MDMs: monocyte-derived macrophages; moDCs: monocyte-derived dendritic cells.
3. **Secretome profiling:** Even without access to a multiplexing assay, the volume of supernatant obtained following cell culture using this platform is sufficient for measuring up to seven different soluble protein mediators with replicates. Different cytokines and chemokines including TNF-α, IL-10, TGF-β1, CCL-18, IL-6, and IL-12 were assayed from the supernatants using high-binding 384-well plate and ELISA DuoSet^®^ kits. A representative example for moDCs and MDMs is shown in Figure 9. The capability to measure various soluble factors using this platform serves as compelling evidence for the exceptional integrity of the fabricated polymer surfaces. Indeed, this indicates that the system is capable of effectively accommodating a diverse range of chemistries.
  **Figure 9.**
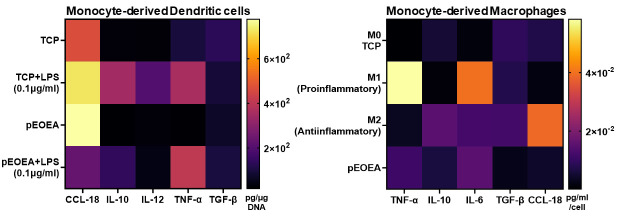
Secretome analysis of two primary cell types after incubation with the pEOEA. Heat maps are the representative mean values of cytokine ELISA results from three independent experiments with two technical repeats. TCP, M0, M1, and M2 are internal controls for comparisons only. TCP: tissue culture plastic control.
**Data Availability Statement:** The data that support the findings of this study are openly available at the University of Nottingham data repository, DOI: 10.17639/nott.7362.
